# Analysis of health risk factors for older adults living alone in China and establishment and evaluation of a nomogram prediction model

**DOI:** 10.3389/fpubh.2024.1309561

**Published:** 2024-03-19

**Authors:** Kexin Chen, Jiangwei Qiu, Wenlong Wang, Qi Hu, Hui Qiao

**Affiliations:** ^1^School of Public Health, Ningxia Medical University, Yinchuan, China; ^2^Key Laboratory of Environmental Factors and Chronic Disease Control, Yinchuan, China

**Keywords:** Chinese general social survey, older adults living alone, health, internet use, mandarin proficiency, nomogram

## Abstract

**Objective:**

To understand the health status of older adults living alone in China and analyze the influencing factors, so as to provide reference for improving the health status of older adults living alone.

**Methods:**

Based on CGSS data from China General Social Survey (2017), the influencing factors of health status of older adults living alone were analyzed by unconditional Logistic regression, and the R software was used to develop a nomogram for predicting the risk of self-assessed unhealthy adverse outcomes.

**Results:**

Gender, annual income, mandarin listening level and participation in medical insurance were the influencing factors of self-rated health of older adults living alone. Age and annual income are the influencing factors of physiological health. Annual income and Internet use were influential factors for mental health. C-Statistic of nomogram prediction model was 0.645. The calibration curve showed that goodness of fit test (*χ^2^* = 58.09, *p* < 0.001), and the overall prediction ability of the model was good.

**Conclusion:**

The health status of older adults living alone in the home-based older adults care is worrying, and it is affected by various factors. We should pay more attention to older adults living alone, improve the ability of listening and distinguishing mandarin and the use of health information platforms for older adults living alone, and further implement medical insurance policies and health services. Announcing the solution to promote healthy home-based care for older adults living alone.

## Introduction

China, as a populous nation, is currently grappling with the profound challenges posed by its aging population. According to the Blue Book on Aging: China Aging Research Report 2022 released by the Chinese Gerontological Society, by the end of 2021, China’s population aged 60 and above reached 267 million, accounting for 18.9% of the total population (1). The fourth survey on the living conditions of urban and rural older adults in China reveals that the number of empty-nest older adults exceeds 100 million, with some major cities and rural areas having a proportion exceeding 70% (2). The transition to smaller family sizes and a preference for traditional care models contribute to a significant increase in the number of isolated older individuals lacking robust social support networks (3). In this context, China, in pursuit of the development goals for achieving Universal Health Coverage (UHC), has prioritized the enhancement of older adults health management (4). This demographic shift towards an aging society necessitates a comprehensive examination of the health and well-being of older adults individuals, particularly those who live alone (5). It is crucial to identify and mitigate health risks among older individuals living alone, providing them with appropriate medical services and support.

Older individuals living alone, separated from their children or other family members, confront numerous challenges, including inconveniences, difficulties, and safety hazards in home-based care (6, 7). A cohort study showed that living alone increases the risk of hypertension in older adults in China (7). Moreover, research has illuminated that living alone, compared to cohabiting with family, is significantly associated with higher levels of depression (8). Previous research has proposed encouraging older individuals living alone to engage in social activities as a means to improve sleep quality, alleviate anxiety, and address the loneliness associated with solitary living (9). Research has shown that older individuals living alone are more susceptible to environmental risks, such as falls, compared to those cohabiting with others (10). Devoid of robust social relationships and assistance, these isolated older adults are susceptible to malnutrition, accidents, self-neglect, untreated medical conditions, and other health threats (11–13).

Former research on the health status of solitary older adults in home-based care has centered largely on disparate regions (7), community care models (14) and support systems (15). While most of these studies addressed disease factors and nutritional status, they devoted less consideration to the connections between the health conditions of solitary seniors and elements like mandarin proficiency, digital literacy, health insurance coverage, and pension benefits, overlooking some critical information. Yet these factors may be key determinants impacting the welfare of this vulnerable group. Research has indicated that digital inclusion and cultural capital contribute to improving residents’ health conditions (16). Digital health literacy is beneficial for enabling older adults to access medical services, participate in social activities, and enhance their quality of life (17). Previous studies have proposed that health insurance and pension assistance can reduce catastrophic health expenditures among older adults (18).

While earlier studies have firmly established connections between physical and mental well-being and factors like income security, insurance coverage, and technical literacy in the general older adults populace, (19–21) targeted investigations into how these relationships distinctly manifest among solitary older adults have been lacking. This study aims to analyze key social determinants impacting the health outcomes of this cohort by delineating the physiological and psychological health challenges and empirical insights into self-rated health faced by solitary older adults. Meanwhile, transforming the intricate regression equations into visualized plots enhances the interoperability of the predictive models, aiding home care workers in appraising solitary seniors. By proactively comprehending the multifaceted risks solitary older adults confront, concrete steps can be taken to prevent deterioration in health and quality of life as this population swells.

## Materials and methods

### Data source and variable definition

The data is from the 2017 Chinese General Social Survey (CGSS) released by Renmin University of China. The 2017 survey data as the latest data for the project. CGSS is the first large-scale, comprehensive, continuous national social survey project in China. The survey samples were selected through multi-stage stratified sampling, covering 28 provinces, autonomous regions and municipalities in China. It is highly representative of the national population with high data quality (22). According to research needs, the target group in this study was set as “solitary older adults aged 60 and above.” After excluding missing values and extreme outliers, the effective total sample size included for analysis was 1,015. The database contains variables including gender, income, internet use, health status, participation in health insurance, and participation in pension insurance.

The dependent variable is the health status of solitary older adults. Based on the WHO’s definition of health from physical, mental and social perspectives as “a state of complete well-being,” the health conditions of solitary older adults were comprehensively examined from three aspects: self-rated health, physical health, and mental health (23). The questionnaire items “How do you rate your current physical health?,” “How often did your health issues affect your work or other regular activities in the past 4 weeks?,” and “How often did you feel depressed or despair in the past 4 weeks?” were used to measure self-rated health, physical health, and mental health of solitary older adults, respectively. The above three items were measured using a 5-point Likert scale. Self-rated health, physical health, and mental health were assigned as binary classification variables. Specifically, self-rated health (very healthy and relatively healthy were assigned 1, general, relatively unhealthy and very unhealthy were assigned 0); physical health and mental health (never and rarely were assigned 1, sometimes, often and always were assigned 0). The independent variables encompassed facets such as gender, age, educational attainment, insurance coverage, internet usage, and competency in mandarin reading and listening comprehension. Table 1 shows the variable assignment table.

**Table 1:** Variable assignment Table.

Variable	Definition
Self-rated health	Yes = 1; No = 0
Physical health	Yes = 1; No = 0
Mental health	Yes = 1; No = 0
Age	Continuous variable
Sex	Male = 1; Female = 2
Urban and rural	Rural residents = 1; urban resident = 2
Education	Primary and below =1; Middle School to High School =2; University degree or above = 4
Annual income (Yuan)	<5,000 = 1, 5,000 ~ 25,000 = 2, >25,000 = 3
Participation in medical security	Yes = 1; No = 0
Participation in pension insurance	Yes = 1; No = 0
Internet usage	Never =1; Rarely =2; Sometimes =3; Often =4; Always =5
Listening level of mandarin	Completely unintelligible =1; poor =2, average = 3; good =4; excellent =5
Speaking level of mandarin	It is completely impossible to say =1; poor =2; average = 3; good =4; excellent =5

### Statistical analyses

Statistical analysis was performed using SPSS 20.0 and R software (version 4.1.1). Quantitative data were described, and count data were expressed as frequency or percentage. Logistic regression analysis was used to analyze influencing factors of the health status of solitary older adults, with a test level of α = 0.05. Based on the results of multivariate logistic regression analysis, a nomogram predicting the risk of poor self-rated health was constructed using the Nomogram function in the rms package of R software. Internal validation was performed using the Bootstrap method with 1,500 resampling, and the C-statistic was calculated and a calibration curve plotted to examine the calibration of the influencing factors model, that is, the consistency between the actual probability of occurrence and the predicted probability of outcomes.

Logistic regression is a widely used method in binary classification problems, and its output is probability, which can be directly interpreted as the likelihood of an event occurring. The probit model is also a viable option, but its output is a probability density function and requires more technical interpretation. In contrast, logistic regression is more intuitive and easy to understand. The tobit model is typically used to deal with truncated data, i.e., where there is a lower or upper bound on the dependent variable. In the health-related variables I studied, there was no such truncation problem. Therefore, logistic regression was used for statistical analysis.

## Results

### Descriptive statistics of the solitary older adults

This study included a total of 1,015 solitary older adults. The oldest was 102 years old and the youngest 60 years old, with an average age of (74.75 ± 8.79) years. There were 463 females (45.6%) and 552 males (54.4%). Regarding marital status, 251 (24.7%) were married and 764 (75.3%) were divorced/widowed/unmarried. In terms of education level, 601 (59.2%) had primary school education or below, 352 (34.7%) had middle school (secondary school) or high school education, and 62 (6.1%) had college degree or above. Regarding household registration status, 521 (51.3%) had rural hukou and 494 (48.7%) had non-rural hukou. For social security programs, 895 (88.2%) participated in basic urban health insurance, new rural cooperative medical care or free medical care (abbreviated as health insurance participation), 120 (11.8%) did not participate; 777 (76.6%) participated in urban or rural basic pension insurance (abbreviated as pension insurance participation), 238 (23.4%) did not participate. For self-rated health, 659 (64.9%) rated themselves as unhealthy and 356 (35.1%) as healthy. For physical health, 479 (47.2%) rated themselves as unhealthy and 536 (52.8%) as healthy. For mental health, 428 (42.2%) rated themselves as unhealthy and 587 (57.8%) as healthy.

### Univariate logistic regression analysis of health status of solitary older adults

Table 2 reports with self-rated health, physical health, and mental health of solitary older adults as dependent variables, and gender, household registration status, education level, annual income, health insurance participation, pension insurance participation, internet use, mandarin listening comprehension level, and mandarin speaking level as independent variables logistic regression analysis was performed. The results showed that gender, household registration status, education level, annual income, health insurance participation, mandarin listening comprehension level, and mandarin speaking level may be influencing factors for self-rated health of solitary older adults. Age, household registration status, education level, annual income, internet use, mandarin listening comprehension level, and mandarin speaking level may be influencing factors for physical health of solitary older adults. Household registration status, education level, annual income, internet use, mandarin listening comprehension level, and mandarin speaking level may be influencing factors for mental health of solitary older adults (*p* < 0.05).

**Table 2:** Univariate logistic regression analysis of health status of older adults people living alone.

Variable	Self-rated health			Physical health			Mental health		
	*β*	*OR*	95%*CI*	*β*	*OR*	95%CI	*β*	*OR*	95%*CI*
Age	−0.01	0.99	0.98 ~ 1.00	−0.03	0.97^**^	0.96 ~ 0.99	0.01	1.01	0.99 ~ 1.02
Sex (ref = male)	0.39	0.68^*^	0.52 ~ 0.87	−0.17	0.85	0.66 ~ 1.08	−0.09	0.92	0.72 ~ 1.18
Urban and rural (ref = urban resident)	0.43	1.54^*^	1.19 ~ 1.20	−0.95	2.60^**^	2.02 ~ 3.35	−0.79	2.21^**^	1.71 ~ 2.85
Education	0.40	1.49^**^	1.21 ~ 1.84	0.62	1.85^**^	1.50 ~ 2.30	0.46	1.58^**^	1.28 ~ 1.96
Annual income (Yuan)	0.41	1.51^**^	1.29 ~ 1.76	0.74	2.10^**^	1.80 ~ 2.47	0.67	1.96^**^	1.67 ~ 2.30
Participation in medical security (ref = no)	−0.44	0.65^*^	0.44 ~ 0.95	−0.29	0.75	0.51 ~ 1.10	−0.26	0.77	0.52 ~ 1.14
Participation in pension insurance (ref = no)	0.13	1.14	0.84 ~ 1.56	0.21	1.24	0.93 ~ 1.26	0.26	1.30	0.97 ~ 1.74
Internet usage	0.02	1.02	0.98 ~ 1.07	0.33	1.39^**^	1.24 ~ 1.57	0.32	1.38^**^	1.22 ~ 1.56
Listening level of mandarin	0.38	1.47^**^	1.29 ~ 1.66	0.36	1.43^**^	1.28 ~ 1.61	0.30	1.36^**^	1.21 ~ 1.52
Speaking level of mandarin	0.26	1.30^**^	1.18 ~ 1.44	0.32	1.37^**^	1.24 ~ 1.52	0.28	1.32^**^	1.19 ~ 1.46

^*^p < 0.05; ^**^p < 0.01.

### Multivariate logistic regression analysis of health status of solitary older adults

Table 3 reports with self-rated health, physical health, and mental health of solitary older adults as dependent variables, and statistically significant indicators from univariate logistic regression analysis as independent variables, multivariate logistic regression analysis was performed. The results showed that gender, annual income, mandarin listening comprehension level, and health insurance participation were independent influencing factors for self-rated health of solitary older adults. Age and annual income were independent influencing factors for physical health of solitary older adults. Annual income and internet use were independent influencing factors for mental health of solitary older adults (*p* < 0.05).

**Table 3:** Multivariate logistic regression analysis of health status of older adults people living alone.

Variable	Self-rated health			Physical health			Mental health		
	*β*	OR	95%*CI*	*β*	OR	95%CI	*β*	OR	95%CI
Age				−0.03	0.97^**^	0.96 ~ 0.99			
Sex (ref = male)	0.41	1.51^**^	1.14 ~ 1.20						
Urban and rural (ref = urban resident)	0.13	1.14	0.76 ~ 1.71	−0.34	0.71	0.49 ~ 1.05	−0.18	0.83	0.57 ~ 1.22
Education	0.01	1.00	0.76 ~ 1.33	−0.15	0.86	0.64 ~ 1.16	−0.24	0.79	0.59 ~ 1.01
Annual income (Yuan)	0.35	1.42^**^	1.13 ~ 1.78	0.60	1.82^**^	1.47 ~ 2.27	0.56	1.76^**^	1.42 ~ 2.18
Participation in medical security (ref = no)	−0.52	0.60^**^	0.39 ~ 0.91						
Participation in pension insurance (ref = no)				0.10	1.10	0.96 ~ 1.27	0.20	1.23^**^	1.06 ~ 1.42
Internet usage	0.29	1.33^**^	1.12 ~ 1.59	0.14	1.16	0.97 ~ 1.37	0.14	1.14	0.97 ~ 1.35
Listening level of mandarin	0.02	1.02	0.86 ~ 1.20	−0.05	0.95	0.80 ~ 1.12	−0.05	0.95	0.81 ~ 1.12

^*^p < 0.05; ^**^p < 0.01.

### Nomogram model building for risk of self-rated unhealthy in solitary older adults

**Figure 1:**
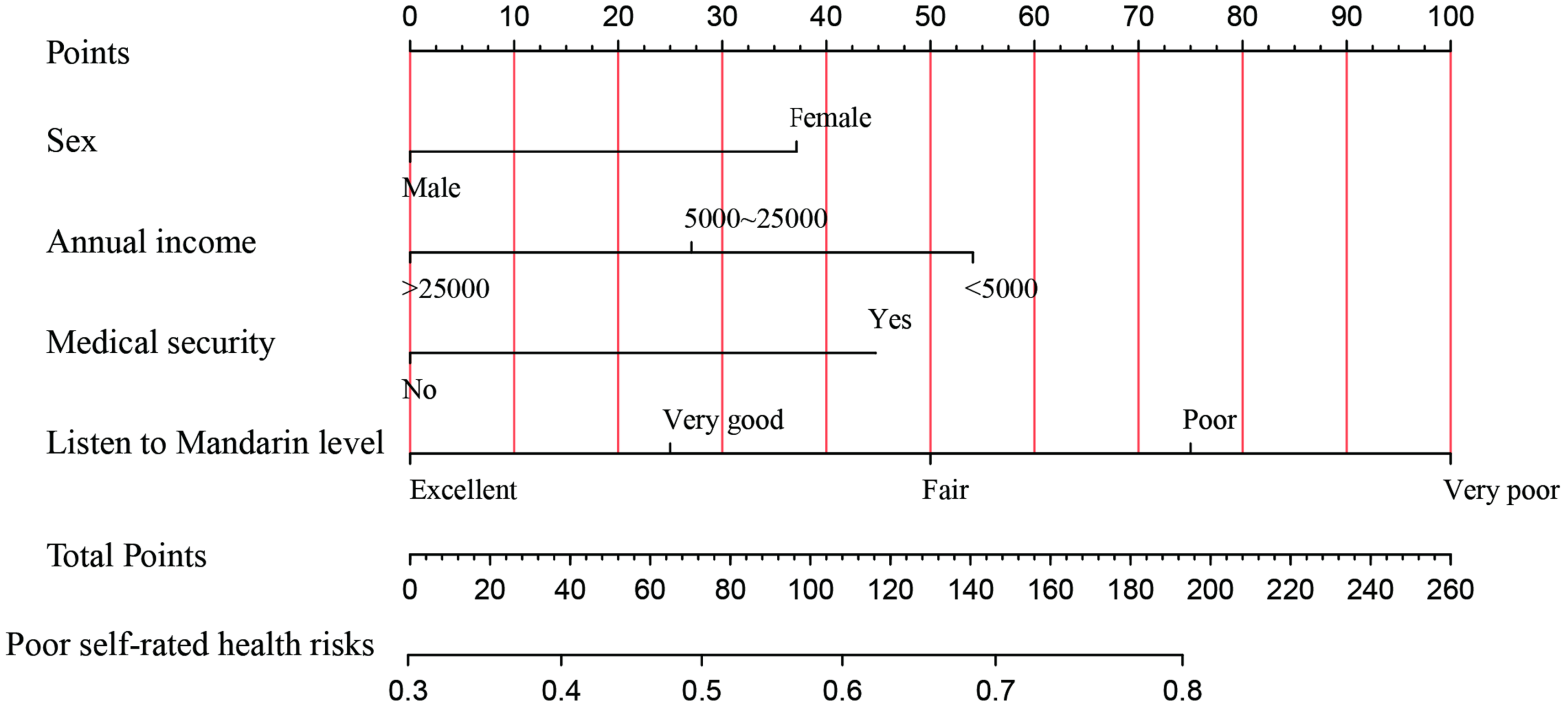
Nomogram model for the risk of self-rated unhealthy in solitary older adults. The different categories of each variable correspond to points on the nomogram axis. The score (points) for each variable can be obtained by drawing a vertical line upwards from the point. The total score (total points) is calculated by adding up the scores for each variable. The total score corresponds to a point on the risk axis for poor outcome, which represents the risk of self-rated unhealthy for the solitary older adult.

Based on the results of multivariate logistic regression analysis, a nomogram model for the risk of self-rated unhealthy was constructed using R software. The nomogram results show that each factor’s value corresponds to the “points” in the first row, and the total points are calculated by adding up the points for each factor. The total points then correspond to the “Risk of Self-Rated Unhealthy” in the last row. If a solitary older adult is female, has an annual income less than 5,000 yuan or between 5,000–25,000 yuan, is uninsured, and has poor mandarin listening comprehension, the corresponding points increase. As the total score from adding up the points for each factor increases, the risk of that solitary older adult self-rating as unhealthy also increases (see Figure 1).

### Validation of the nomogram model for self-rated unhealthy risk in solitary older adults

**Figure 2:**
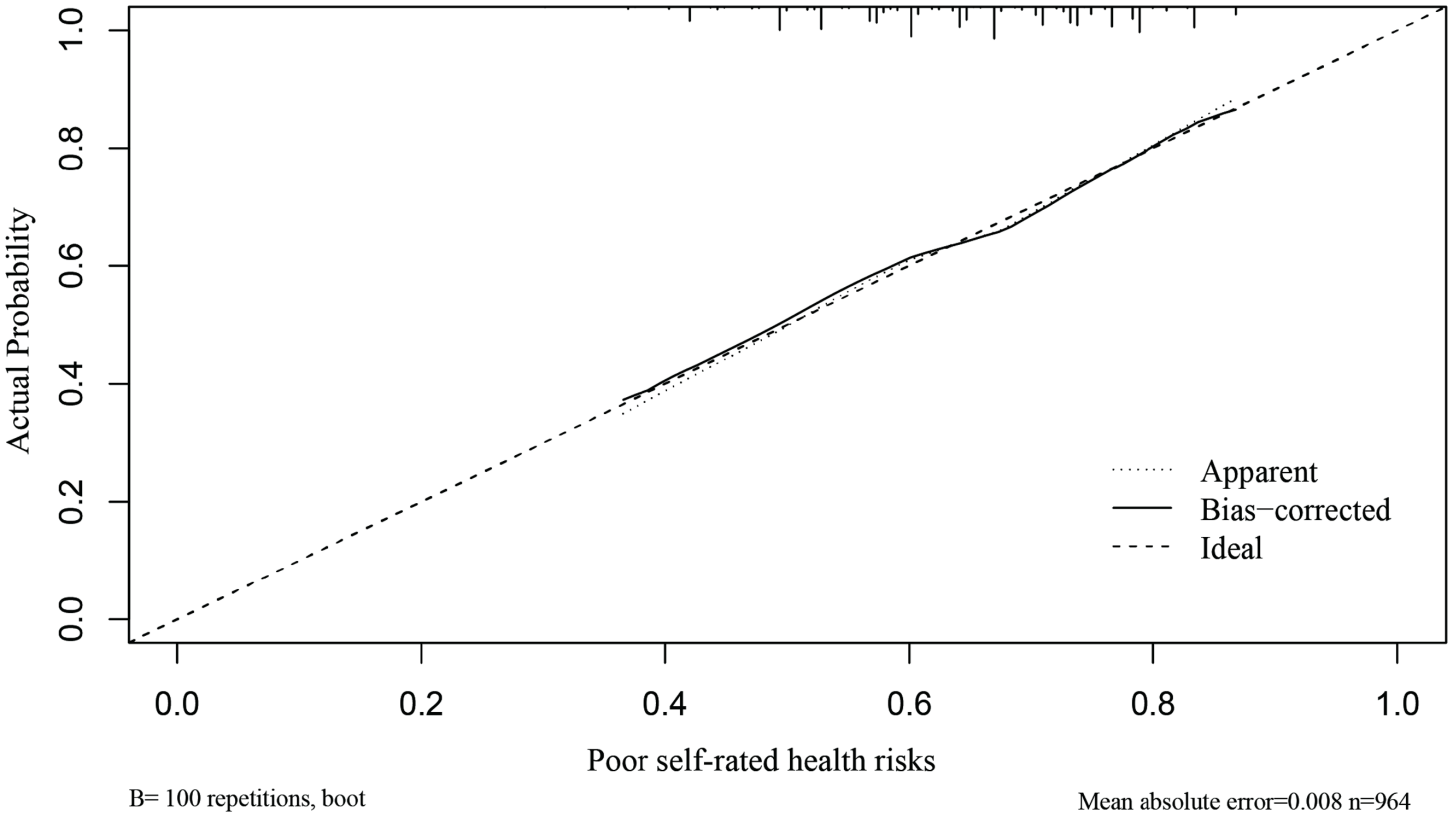
Calibration curve for the nomogram model predicting the risk of self-rated unhealthy in solitary older adults. The diagonal line represents the ideal curve, indicating agreement between actual and predicted values. The dotted line represents the original curve, and the solid line represents the calibrated curve. The closer to the ideal curve, the better the model’s predictive ability.

The C-index and calibration curves were used to evaluate the nomogram model. A C-index value closer to 1 indicates stronger predictive ability of the model. Internal validation via bootstrapping with 1,500 resamples was performed for this nomogram model (C-index = 0.645). The goodness of fit test gave *χ^2^* = 58.09, *p* < 0.001, indicating that the established nomogram model has good accuracy, discrimination, and risk prediction capability (see Figure 2).

## Discussion

The results of this study show that solitary older adults have poor health conditions. Among the 1,015 solitary older adults, 64.9% (659 people) rated their current physical health as unhealthy, while 35.1% (356 people) self-rated as healthy. For physical health, 47.2% of solitary older adults (479 people) self-rated as unhealthy and 52.8% (536 people) as physically healthy. For mental health, 42.2% of solitary older adults (428 people) self-rated as mentally unhealthy and 57.8% (587 people) as mentally healthy.

Self-rated health reflects individuals’ perceptions of their own health status (24). The results of this study show that gender, annual income, mandarin comprehension level, and participation in basic urban/rural health insurance programs are factors influencing self-rated health of solitary older adults. Females had poorer self-rated health compared to males. Higher income is a protective factor for self-rated health in solitary older adults, likely because older adults with higher income have more freedom in spending on healthy aging and medical care, which facilitates adhering to healthy behavioral measures (25, 26). Solitary older adults with better mandarin comprehension had higher self-rated health, possibly due to the increasing incidence of age-related hearing impairment over the years (27). Meanwhile, the popularity of mandarin is relatively low among older adults population, and speech recognition ability also impacts cognitive function in older adults (28). The older adults living alone who were covered by medical insurance were more likely to have better self-rated health, highlighting the importance of health insurance in UHC (29, 30).

By translating the results of multivariate regression analysis into a nomogram model, we present an intuitive individualized risk assessment approach, aiding in the more precise identification of vulnerable groups that require focused attention in the context of UHC. For instance, the nomogram can be used to promptly identify solitary older adults with higher risk of self-rated unhealthy. If a female older adult has an annual income of 5,000–25,000 yuan, participates in health insurance, and has poor mandarin comprehension, her points for each variable would be 37, 27, 45, and 75, respectively, with a total score of 184. This corresponds to 0.79 on the nomogram, indicating a 79% risk of self-rated unhealthy. For solitary older adults at higher specific risks, community health workers can employ information technology to deliver targeted interventions, thereby promoting healthy aging (31).

The physical health of solitary older adults, considered as a pivotal component in the measurement of multidimensional health, serves as a prominent predictor of overall health and mortality risk (32). Previous studies show that physical health is influenced by demographic and social characteristics, lifestyle, physical health functions, social health, and economic status (33). This study indicates advanced age and low income are independent factors influencing physical health of solitary older adults, with worse physical health at older ages. Income level also determines physical health, with lower-income solitary older adults having poorer physical health. This may be attributable to the irreversible decline in bodily structure and function that accompanies the march of senescence. Household economic status impacts the proportion of medical expenses in total consumption expenditures. Insufficient income cannot ensure basic medical security (30). Coupled with higher rates of chronic diseases in solitary older adults, lack of medical support greatly reduces their physical health (34).

As solitary living becomes more prevalent among older adults in China, the emotional changes and coping abilities of older adults facing long-term isolation warrant close attention. The results of this study reveal concerning mental health conditions among solitary older adults. Their psychological well-being is associated with increased income and internet use. This may be because older adults with higher economic status can fulfill basic needs for food, shelter, and clothing, then pursue higher-level needs of healthy social and recreational activities to safeguard mental health, effectively alleviating feelings of helplessness and loneliness from loss of a spouse or children leaving home, and elevating positive emotions (7, 34). Additionally, greater income implies solitary older adults can access and utilize more medical resources, reducing mental health risks. Internet use also clearly benefits the psychological well-being of solitary older adults, aligning with findings by Zhao et al. (35). The internet provides solitary older adults platforms for communication and learning, fulfilling the need for continued socialization (20). It helps to adapt to the changes in roles after retirement, increase social activities and intra-family decision-making, and reduce psychological discomfort in older adults (36, 37).

Within the framework of UHC, we advocate for greater attention to older people living alone by providing more pension assistance and health support. Family doctors and community nurses should be encouraged to adopt informational means to deliver home-based older adults care health services, penetrate into solitary older adults, guide older adults toward proactive aging approaches, improve their health service utilization, and provide government assistance and community public health services to economically disadvantaged solitary older adults. Confronting healthy aging, medical insurance functions should be fully exercised to implement policy publicity, promoting awareness of medical insurance compensation policies among solitary older adults. Meanwhile, relevant departments could jointly carry out promotion activities on mandarin proficiency and smartphone use to enhance solitary older adults’ mandarin comprehension and health knowledge exchange abilities. This can lower barriers and fears toward medical and health information platforms, increase adoption of “Internet Plus” healthcare among solitary older adults, strengthen interpersonal communication and health knowledge learning capabilities. At the same time, easy-to-understand online health lectures could be organized, and remote chronic disease management, physical examinations, psychological counseling and other older adults care health services provided, to facilitate healthy home-based aging among solitary older adults.

While our study benefits from a nationwide data set, it is crucial to acknowledge certain limitations. This research relies on cross-sectional data, and although it identifies health risk factors among solitary older individuals, it does not delve into the complex relationships between these factors. Future research endeavors may consider adopting a longitudinal study design to comprehensively understand the evolution of health conditions among solitary older individuals over time and to identify enduring influencing factors. This approach can contribute to the development of more effective intervention measures and policies.

## Conclusion

We utilized data from the China General Social Survey (CGSS) to examine health risk factors among solitary older adults in home-based care in China. The research findings indicate that gender, annual income, Mandarin language proficiency, and participation in health insurance significantly influence the self-rated health of solitary older individuals. Age and annual income emerge as factors affecting their physical health, while income and internet usage are identified as influencing factors on their psychological well-being. Furthermore, based on the results of multivariate logistic regression analysis, we constructed a line chart model for assessing the risk of self-assessed poor health. The evaluation of the model demonstrates its favorable accuracy, discriminative power, and risk prediction capability.

## Data availability statement

The original contributions presented in the study are included in the article/supplementary materials, further inquiries can be directed to the corresponding authors.

## Author contributions

KC: Conceptualization, Data curation, Formal analysis, Methodology, Writing – original draft. JQ: Conceptualization, Data curation, Formal analysis, Methodology, Writing – original draft. WW: Conceptualization, Methodology, Project administration, Writing – original draft. QH: Conceptualization, Methodology, Funding acquisition, Writing – review & editing. HQ: Conceptualization, Funding acquisition, Resources, Supervision, Writing – review & editing.

## References

[1] Wang H Chen H. Aging in China: challenges and opportunities. CCDCW. (2022) 4:601–2. doi: 10.46234/ccdcw2022.130, PMID: 35919296 PMC9339359

[2] Jiang H Xiao S Hu H He H. Study on the measurement and influencing factors of care service demand of disabled elderly in urban and rural China. Int J Environ Res Public Health. (2022) 19. doi: 10.3390/ijerph191711112, PMID: 36078829 PMC9518346

[3] Holt-Lunstad J. The potential public health relevance of social isolation and loneliness: prevalence, epidemiology, and risk factors. Public Policy & Aging Report. (2018) 27:127–30. doi: 10.1093/ppar/prx030

[4] Fang G Yang D Wang L Wang Z Liang Y Yang J. Experiences and challenges of implementing universal health coverage with China's National Basic Public Health Service Program: literature review, regression analysis, and insider interviews. JMIR Public Health Surveill. (2022) 8:e31289. doi: 10.2196/31289 35867386 PMC9356336

[5] Tu WJ Zeng X Liu Q. Aging tsunami coming: the Main finding from China's seventh National Population Census. Aging Clin Exp Res. (2022) 34:1159–63. doi: 10.1007/s40520-021-02017-4, PMID: 34727357

[6] Ruan J Zheng W Zhuang Y. Everyday life experiences of Chinese community-dwelling oldest old who live alone at home. Int J Qual Stud Health Well Being. (2023) 18:2253937. doi: 10.1080/17482631.2023.2253937, PMID: 37667880 PMC10481758

[7] Wang X Yuan X Xia B He Q Jie W Dai M. Living alone increases the risk of hypertension in older Chinese adults: a population-based longitudinal study. Innov Aging. (2023) 7:igad071. doi: 10.1093/geroni/igad071, PMID: 37502337 PMC10370894

[8] Huang M Liu K Liang C Wang Y Guo Z. The relationship between living alone or not and depressive symptoms in older adults: a parallel mediation effect of sleep quality and anxiety. BMC Geriatr. (2023) 23:506. doi: 10.1186/s12877-023-04161-0, PMID: 37608361 PMC10463962

[9] Renren Li LH Shiyue X He F Liu T. Group work practice on alleviating loneliness of elderly living alone in urban areas. Law Soc. (2021) 2:109–10. doi: 10.19387/j.cnki.1009-0592.2021.01.141

[10] Lee H Lim JH. Living alone, environmental hazards, and falls among U.S. older adults. Innov Aging. (2023) 7:igad055. doi: 10.1093/geroni/igad055, PMID: 37583969 PMC10424630

[11] Wei K Nyunt MSZ Gao Q Wee SL Ng TP. Long-term changes in nutritional status are associated with functional and mortality outcomes among community-living older adults. Nutrition. (2019) 66:180–6. doi: 10.1016/j.nut.2019.05.006, PMID: 31310959

[12] Liu N Andrew NE Cadilhac DA Yu X Li Z Wang J. Health-related quality of life among elderly individuals living alone in an urban area of Shaanxi Province, China: a cross-sectional study. J Int Med Res. (2020) 48:300060520913146. doi: 10.1177/0300060520913146, PMID: 32253961 PMC7140192

[13] Bu F Abell J Zaninotto P Fancourt D. A longitudinal analysis of loneliness, social isolation and falls amongst older people in England. Sci Rep. (2020) 10:20064. doi: 10.1038/s41598-020-77104-z, PMID: 33303791 PMC7730383

[14] Yi YM Park YH Cho B Lim KC Jang SN Chang SJ. Development of a community-based integrated service model of health and social Care for Older Adults Living Alone. Int J Environ Res Public Health. (2021) 18:825. doi: 10.3390/ijerph18020825, PMID: 33478027 PMC7835935

[15] Zhou R Cui J Yin X. Perceived family relationships and social participation through sports of urban older adults living alone: an analysis of the mediating effect of self-respect levels. Front Public Health. (2023) 11:1095302. doi: 10.3389/fpubh.2023.1095302, PMID: 37064670 PMC10098356

[16] Sun Z Sun W Gao H Fa R Chen S Qian D. Digital inclusion, cultural capital, and health status of urban and rural residents: an empirical study based on 2017 Cgss database. Int J Environ Res Public Health. (2023) 20:4022. doi: 10.3390/ijerph20054022 36901033 PMC10002041

[17] Liu S Lu Y Wang D He X Ren W Kong D. Impact of digital health literacy on health-related quality of life in Chinese community-dwelling older adults: the mediating effect of health-promoting lifestyle. Front Public Health. (2023) 11:1200722. doi: 10.3389/fpubh.2023.1200722, PMID: 37415711 PMC10321557

[18] Zhou Y Wushouer H Vuillermin D Guan X Shi L. Does the universal medical insurance system reduce catastrophic health expenditure among middle-aged and elderly households in China? A longitudinal analysis. Eur J Health Econ. (2021) 22:463–71. doi: 10.1007/s10198-021-01267-3 33582893

[19] Yang L Wang L Di X Dai X. Utilisation of community care services and self-rated health among elderly population in China: a survey-based analysis with propensity score matching method. BMC Public Health. (2021) 21:1936. doi: 10.1186/s12889-021-11989-x, PMID: 34696767 PMC8546940

[20] Du X Liao J Ye Q Wu H. Multidimensional internet use, social participation, and depression among middle-aged and elderly Chinese individuals: Nationwide cross-sectional study. J Med Internet Res. (2023) 25:e44514. doi: 10.2196/44514, PMID: 37647119 PMC10500359

[21] Yang K Li Y Qi H. Determinants of and willingness to use and pay for digital health technologies among the urban elderly in Hangzhou, China. Risk Manag Healthc Policy. (2023) 16:463–78. doi: 10.2147/rmhp.S393767, PMID: 37007299 PMC10064872

[22] Wang H Yang Y You Q Wang Y Wang R. Impacts of physical exercise and media use on the physical and mental health of people with obesity: based on the CGSS 2017 survey. Healthcare. (2022) 10:1740. doi: 10.3390/healthcare10091740, PMID: 36141352 PMC9498912

[23] Wang S Zhang C Xu W. Mindfulness, mortality, disability rates, physical and mental health among the oldest old. Health Psychol. (2023) 42:746–55. doi: 10.1037/hea0001315, PMID: 37616101

[24] Kang W Malvaso A. Self-rated health (Srh) partially mediates and associations between personality traits and life satisfaction in older adults. Front Psychol. (2023) 14:1189194. doi: 10.3389/fpsyg.2023.1189194, PMID: 37484078 PMC10359495

[25] Xin Y Ren X. The impact of family income on body mass index and self-rated health of illiterate and non-illiterate rural elderly in China: evidence from a fixed effect approach. Front Public Health. (2021) 9:722629. doi: 10.3389/fpubh.2021.722629, PMID: 34604161 PMC8484635

[26] Jun J Park D. Factors associated with self-rated health among Korean elderly. Iran J Public Health. (2023) 52:360–70. doi: 10.18502/ijph.v52i2.11889, PMID: 37089149 PMC10113570

[27] Moser S Luxenberger W Freidl W. The influence of social support and coping on quality of life among elderly with age-related hearing loss. Am J Audiol. (2017) 26:170–9. doi: 10.1044/2017_aja-16-0083, PMID: 28445580

[28] Fu X Liu B Wang S Eikelboom RH Jayakody DMP. The relationship between hearing loss and cognitive impairment in a Chinese elderly population: the baseline analysis. Front Neurosci. (2021) 15:749273. doi: 10.3389/fnins.2021.749273, PMID: 34899159 PMC8662817

[29] Zhou Y Wushouer H Vuillermin D Ni B Guan X Shi L. Medical insurance and healthcare utilization among the middle-aged and elderly in China: evidence from the China health and retirement longitudinal study 2011, 2013 and 2015. BMC Health Serv Res. (2020) 20:654. doi: 10.1186/s12913-020-05522-w, PMID: 32664947 PMC7362522

[30] Guets W Behera DK. Does disability increase Households' health financial risk: evidence from the Uganda demographic and health survey. Glob Health Res Policy. (2022) 7:2. doi: 10.1186/s41256-021-00235-x, PMID: 34983699 PMC8728967

[31] Ohta S Nakamoto H Shinagawa Y Tanikawa T. A health monitoring system for elderly people living alone. J Telemed Telecare. (2002) 8:151–6. doi: 10.1177/1357633x0200800305 12097176

[32] Zanocchi M Maero B Francisetti F Giona E Nicola E Margolicci A. Multidimensional assessment and risk factors for prolonged hospitalization in the elderly. Aging Clin Exp Res. (2003) 15:305–9. doi: 10.1007/bf03324514, PMID: 14661821

[33] Sun J Lyu S Zhao R. Socioeconomic inequality in health outcomes among the elderly: evidence from a cross-sectional study in China. Risk Manag Healthc Policy. (2020) 13:397–407. doi: 10.2147/rmhp.S248019, PMID: 32523387 PMC7234974

[34] Xu S Yang X Liu J Chong MK Cheng Y Gong W. Health and wellbeing among the empty Nest and non-empty Nest elderly in China-results from a National Cross-Sectional Study. PLoS One. (2023) 18:e0291231. doi: 10.1371/journal.pone.0291231, PMID: 37699029 PMC10497119

[35] Çiftci N Yıldız M Yıldırım Ö. The effect of health literacy and health empowerment on quality of life in the elderly. Psychogeriatrics. (2023) 23:609–20. doi: 10.1111/psyg.12969, PMID: 37186342

[36] Xu J Zhang Q. The relationship between internet use and mental health of the elderly: analysis of the differences between urban and rural. PLoS One. (2023) 18:e0280318. doi: 10.1371/journal.pone.0280318, PMID: 36701394 PMC9879507

[37] Cv I Behera DK Dash U. Participation of older adults in the intra-household decision-making activities: evidence from the longitudinal ageing study in India. The Journal of Adult Protection. (2021) 23:325–36. doi: 10.1108/JAP-03-2021-0013

